# Epithelioid Mesothelioma Patients with Very Long Survival Display Defects in DNA Repair

**DOI:** 10.3390/cancers15174309

**Published:** 2023-08-29

**Authors:** Monica Ganzinelli, Federica Guffanti, Anna Ianza, Navid Sobhani, Sergio Crovella, Fabrizio Zanconati, Cristina Bottin, Marco Confalonieri, Stefano Fumagalli, Alessandra Guglielmi, Daniele Generali, Giovanna Damia

**Affiliations:** 1 Unit of Thoracic Oncology, Department of Medical Oncology, Fondazione IRCCS Istituto Nazionale dei Tumori, 20133 Milan, Italy; monica.ganzinelli@istitutotumori.mi.it; 2 Laboratory of Preclinical Gynecological Oncology, Department of Experimental Oncology, Istituto di Ricerche Farmacologiche Mario Negri IRCCS, 20156 Milan, Italy; federica.guffanti@marionegri.it; 3 Oncology Department, University Health Organization Giuliano Isontina, ASUGI, Piazza Ospitale 1, 34129 Trieste, Italy; anna.ianza@asuits.sanita.fvg.it (A.I.); alessandra.guglielmi@asugi.sanita.fvg.it (A.G.); daniele.generali@gmail.com (D.G.); 4 Department of Medical, Surgical and Health Sciences, University of Trieste, 34139 Trieste, Italy; navidsobhani19@gmail.com (N.S.); f.zanconati@fmc.units.it (F.Z.); cbottin@units.it (C.B.); marco.confalonieri@asugi.sanita.fvg.it (M.C.); 5 IRCCS Burlo Garofolo, Via dell’Istria 65/1, 34137 Trieste, Italy; crovelser@gmail.com; 6 Laboratory of Biology of Neurodegenerative Disorders, Department of Neuroscience, Istituto di Ricerche Farmacologiche Mario Negri IRCCS, 20156 Milan, Italy; stefano.fumagalli@marionegri.it

**Keywords:** mesothelioma, DNA repair, RAD51 foci, BRCA foci

## Abstract

**Simple Summary:**

DNA repair has an important role in malignant pleural mesothelioma tumorigenesis and progression. The prognosis of mesothelioma patients is very poor and predictive biomarkers are needed for better management. We analyzed the expression of more than 700 genes involved in different cellular pathways using Nanostring technology in a cohort of 54 epithelioid malignant pleural mesothelioma patients. The median survival time of the cohort was 16.9 months and this cut-off was used to classify patients as long and short survivors (LS/SS) with, respectively, an overall survival ≥ and <16.9 months, as well as very long and very short survivors (VLS/VSS) with an overall survival ≥ than 33.8 and < than 8.45 months. A down-regulation of the DNA damage response pathway was found in LS versus SS. These data were validated by the finding that VLS had a lower number of RAD51- and BRCA1-positive tumor cells than VSS. If these data can be corroborated, an easy and cost-effective test could be routinely used to better manage epithelioid malignant pleural mesothelioma patients.

**Abstract:**

Aim: DNA repair has an important role in malignant pleural mesothelioma (MPM) tumorigenesis and progression. Prognostic/predictive biomarkers for better management of MPM patients are needed. In the present manuscript, we analyzed the expression of more than 700 genes in a cohort of MPM patients to possibly find biomarkers correlated with survival. Methods: A total of 54 MPM patients, all with epithelioid histology, whose survival follow-up and formalin-fixed paraffin-embedded tumors were available, were included in the study. Gene expression profiles were evaluated using a Nanostring platform analyzing 760 genes involved in different cellular pathways. The percentages of proliferating tumor cells positive for RAD51 and BRCA1 foci were evaluated using an immunofluorescence assay, as a readout of homologous recombination repair status. Results: Patient median survival time was 16.9 months, and based on this value, they were classified as long and short survivors (LS/SS) with, respectively, an overall survival ≥ and <16.9 months as well as very long and very short survivors (VLS/VSS) with an overall survival ≥ than 33.8 and < than 8.45 months. A down-regulation in the DNA damage/repair expression score was observed in LS and VLS as compared to SS and VSS. These findings were validated by the lower number of both RAD51 and BRCA1-positive tumor cells in VLS as compared to VSS. Conclusions: The down-regulation of DNA repair signature in VLS was functionally validated by a lower % of RAD51 and BRCA1-positive tumor cells. If these data can be corroborated in a prospective trial, an easy, cost-effective test could be routinely used to better manage treatment in MPM patients.

## 1. Introduction

Malignant pleural mesothelioma (MPM) is an aggressive tumor originating from the mesothelium [1]. Its incidence is still increasing in Europe due to the long latency time, ranging from 20 to 40 years, and persistent asbestos exposure [2]. Histologically, three subtypes can be recognized: the epithelioid, the most frequent; the sarcomatoid; and the biphasic histotype. Systemic chemotherapy treatments have been demonstrated to improve survival in randomized trials; surgery and/or radiotherapy are used, but their role is still debated [3]. Very recently, ipilimumab and nivolumab have received FDA approval for the first-line treatment of unresectable MPM [4]. Nevertheless, the prognosis of these patients is still very poor, with a survival time of 9–18 months [1]. 

DNA repair has an important role in MPM tumorigenesis and progression [5,6]. TCGA analysis of 82 MPM samples revealed that the frequency of one somatic variant in at least one gene involved in the DNA damage response is higher than the overall population [7]. Germline mutations in *BAP1* (*BRCA1-associated protein* gene) were among the first associated with an increased risk of developing MPM [8], followed by other candidate genes involved in DNA repair pathways, including homologous recombination repair (HR) pathways (i.e., *BRCA2* and *MRE11A*) [9,10,11]. 

While mutational inactivation of DNA repair genes renders mesothelial cells more prone to accumulate DNA damage and possibly to malignant transformation, they could also predict a better response to therapy, as observed in other tumor types, i.e., ovarian carcinomas [12]. It has been clearly demonstrated how the deficiency in DNA repair pathways renders tumor cells specifically susceptible to cytotoxic and targeted agents. The most important example is the synthetic lethality between homologous recombination (HR) deficiency (due to mutations in *BRCA1* and *BRCA2* genes) and poly-(ADP-ribose) polymerase inhibitors (PARPi), which have changed the treatment of ovarian cancer patients and significantly improved their prognosis [13]. The sequence of 198 mesothelioma tumors revealed that 55% carried germline and/or somatic mutations in genes involved in the HR repair, which were shown to have both prognostic and predictive roles [14]. These data have opened up the testing of PARPi in this patient subset. Indeed, two phase II clinical trials have been published on the efficacy of PARP inhibitors in MPM [15,16]. Ghafoor et al. [15] reported limited olaparib activity in refractory mesothelioma patients, with shorter progression free survival and overall survival (OS) in patients harboring *BAP1* mutations. Fennell et al. [16] reported some activity of rucaparib in mesothelioma patients with *BAP1* and *BRCA2* alterations. However, in both studies, a limited number of patients have been enrolled (23 and 26, respectively) and included both pleural and peritoneal mesothelioma patients, refractory [15], or relapsing after the first-line therapy [16] supporting further clinical investigation. 

Longer survival was reported in MPM patients with germline mutations in DNA repair genes treated with a platinum-based therapy as compared to those with no mutations [7,17,18], likely based on the role of DNA repair in platinum agents’ cytotoxicity. *BAP1* mutations have not only been reported to be associated with less aggressive tumors and increased in OS [18], but have also been suggested to predict response to immunotherapy [19]. 

RAD51 is a key protein in HR repair [20] and its foci induction has been considered a readout of a functional HR repair [21]. However, we have recently reported how the basal number of RAD51 foci number in proliferating (geminin positive) tumor cells (RAD51 foci score), detected in formalin-fixed paraffin-embedded (FFPE) tumor samples using an immunofluorescence assay, correlated with tumor HR deficiency status and was predictive of olaparib response in an ovarian xenobank; RAD51 foci score also predicted the response to platinum-based therapy in breast cancer [22,23]. These data support RAD51 foci score as a functional biomarker of HR repair.

In the present manuscript, we analyzed the expression of more than 700 genes using the Nanostring^®^ platform in a cohort of 54 MPM patients and correlated the results with patients’ overall survival. We found that long and very long MPM patient survivors displayed a decreased DNA repair expression profile as compared to short and very short MPM patient survivors and these data were functionally confirmed by a much lower RAD51 score and BRCA1 score in very long as compared to very short survivors. 

## 2. Methods

### 2.1. Patients Cohort

This is a retrospective study aimed at identifying possible biomarkers associated with OS in 54 epithelioid MPM patients, all with epithelioid histology, whose formalin-fixed, paraffin-embedded (FFPE) tumor slides were retrieved from the Pathology Unit of the Department of Medical, Surgical and Health Sciences, University of Trieste (Italy). This study included MPM patients treated at the Azienda Sanitaria Universitaria Giuliano Isontina of Trieste between 2006 and 2018. The inclusion criteria were the availability of sufficient tumor material and OS information determined from initial diagnosis until death or loss to follow-up. Tissue samples were collected at diagnosis, prior to any systemic treatment. The protocol and all amendments were approved by the “Comitato etico unico regionale del Friuli Venezia Giulia” (CEUR FVG) (authorization # 0029379/P /GEN/ARCS). The present study was conducted in accordance with the International Conference on Harmonization Guidelines on Good Clinical Practice and the Declaration of Helsinki. 

### 2.2. RNA Isolation from FFPE Tumor Samples and Gene Expression Analysis

Tumor content in FFPE samples was >70%. RNA isolation was performed by using the Maxwell RSC RNA FFPE Kit (Promega, Madison, WI, USA) and its concentration was determined using the NanoDrop spectrophotometer (Thermo Scientific, Waltham, MA, USA). Gene expression analysis was performed using the NanoString nCounter Gene Expression Platform and analyzed with the nCounter system, normalized using R (NanostringNorm R package; version 1.2.0, http://cran.r-project.org/=NanoStringNorm) (accessed on 20 August 2023) [24]. Briefly, RNA was hybridized overnight at 67 °C and the hybridization reactions introduced in the Prep Station, a liquid handling robot for the purification of the hybridized complexes and the immobilization onto the surface of a cartridge. Each sample was identified with a barcode and counted using the Digital Analyzer. The resulting data were imported into and analyzed using the nSolver Analysis Software System 4.0. The nSolver default settings were used to set the quality control parameters recommended by NanoString. Samples that failed the quality control metrics were excluded from further analysis. For data normalization, raw counts were adjusted by background and by internal negative controls followed by a within-sample normalization using the internal positive controls. Finally, data were normalized across samples (i.e., corrected for input) using the mean RNA counts from reference (housekeeping) genes.

### 2.3. Quantitative Real-Time PCR

Real-Time PCRs (RT-PCR) were performed to validate gene expression findings. RNA samples were retro-transcribed with the High-Capacity cDNA Reverse Transcription kit (Applied Biosystems, Waltham, MA, USA). Gene expression levels were measured using quantitative RT-PCR with SYBR green technology (Applied Biosystems, USA) using ad hoc-designed primers (Supplementary Table S1). The cDNA samples were run in triplicate. All data were normalized to the levels of the actin gene and analyzed using the DDCt method. 

### 2.4. Immunofluorescence (IF) Detection of Nuclear Foci on FFPE OC-PDX Samples

RAD51 and γH2AX nuclear foci were quantified as already published [22]. BRCA1 foci were detected following the same protocol for RAD51 foci. Briefly, FFPE tumor tissue sections were deparaffinized and antigens were retrieved with DAKO Antigen Retrieval Buffer pH 9.0 and incubated with primary and secondary antibodies (The full list of the antibodies used and their conditions are reported in Supplementary Table S2). Nuclei were stained with 4′,6-diamidino-2-phenylindole (DAPI) (30 ng/mL in PBS) (Sigma–Aldrich, Burlington, MA, USA). Slides were mounted with Vectashield solution (VectorLab, Sorrento, Italy). Slices were observed using the ECLIPSE Ti2-E fluorescence microscope (Nikon, Tokyo, Japan), with the 60×/1.27 WI Plan APO IR, ∞ 0.15/0.19 WD 0.18–0.16 objective (Nikon). RAD51/BRCA1/γH2AX foci were quantified by scoring in blind the percentage of geminin-(GMN) positive tumor cells with 5 or more foci per nucleus. At least 100 GMN positive cells in three or more different areas of the tissue section were analyzed. 

## 3. Results 

### 3.1. Patients Cohort 

All patients had pathologically confirmed pleural mesothelioma and their clinical characteristics are reported in Table 1. All the tumors were epithelioid. The median age at diagnosis was 73 years; most of the patients were male; occupational or para-occupational information as well as asbestos exposure or smoking exposure information were not available for most of them. Supplementary Figure S1 shows the OS of the entire patient population under study and Supplementary Table S3 reports the descriptive statistic of the patients’ cohort. Median patient survival time was 16.9 months. Based on the median OS, we classified patients as long survivors -LS- (OS ≥ than 16.9) and short survivors -SS- (OS < than 16.9 months); in addition, very long -VLS- and very short -VSS- survivors were defined, respectively, as those with an OS ≥ than 33.8 months (twice the median of the cohort) and with an OS < than 8.45 months (half of the median). This second classification allowed the identification of the most different groups in terms of survival outcome (VSS versus VLS). LS were younger than SS (68.4 years ± 1.8 vs. 73.9 years ± 1.2, *p* < 0.0141), while no difference in ages was found between VSS and VLS groups (73 ± 7 vs. 68 ± 7.6, *p* = 0.22). All the different groups were quite balanced for surgery and chemo/radiotherapy treatments (Supplementary Table S4). Unfortunately, we were not able to retrieve the information of what type of chemotherapy the patients received. However, based on the Italian treatment guidelines [25], it is likely that most patients underwent platinum-based therapy, as specified in Supplementary Table S4. 

### 3.2. Nanostring Gene Expression

**Figure 1:**
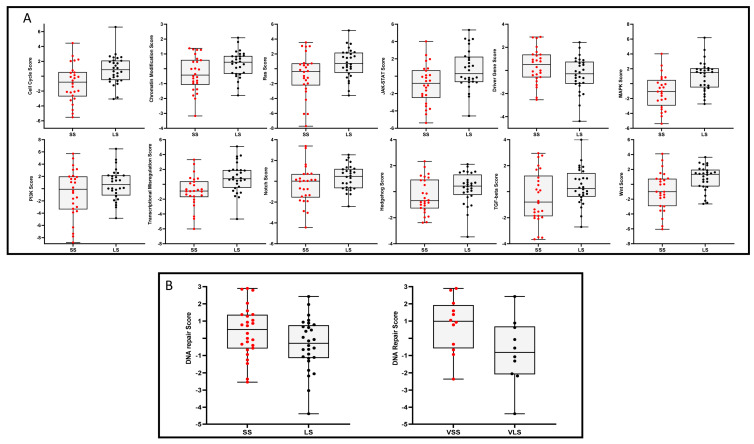
Panel (**A**) Nanostring signature scores in individual short (SS) and long (LS) survivors MPM patients analyzed. Each box represents a different pathway under study and each dot represents an individual patient score. Panel (**B**) Left panel: DNA damage repair (DDR) score in SS and LS survivors. Right panel: DDR score in very short (VSS) and very long (VLS) survivors. Each dot represents an individual patient score.

Gene expression profiles were evaluated using the Nanostring platform that includes 760 genes involved in different cellular pathways. We first looked for genes that were differentially expressed between SS and LS. Supplementary Figure S2A reports the Volcano plot data and Supplementary Figure S2B shows the results of the RT-PCR performed to validate the genes that were differentially expressed in the two groups of patients. We found higher NR4A1, NR4A3, GRIN2A, and FOS mRNA levels in LS as compared to SS. These genes were associated with survival using univariate analysis; however, these data could not be confirmed using multivariate analysis (Supplementary Table S5). We then looked for 13 cellular pathways differentially evaluated using the Nanostring algorithm and reported in Figure 1A; all the pathways considered showed a trend over an upregulation in LS as compared to SS patients, except for the DNA damage repair (DDR) pathway that was downregulated in LS (Figure 1B, left panel). The DDR pathway consists of 44 genes, including *ATM*, *ATR*, *BRCA1*, *BRCA2*, *CHECK1*, *CHECK2*, *FANCA*, *FANCD2*, *MLH1*, *MSH2*, *PARP1*, *PARP2*, *PARP3*, and *RPA3*. We further analyzed these DDR scores in the subgroups of VSS (median OS < 8.45 months) and VLS (median OS ≥ 33.8) confirming the previous observation (Figure 1B, right panel). Supplementary Table S6 reports the expression levels of all the genes considered for the evaluation of the DNA damage score in VLS as compared to VSS.

### 3.3. Functional Characterization of DNA Repair Status in MPM Tumor Samples

**Figure 2:**
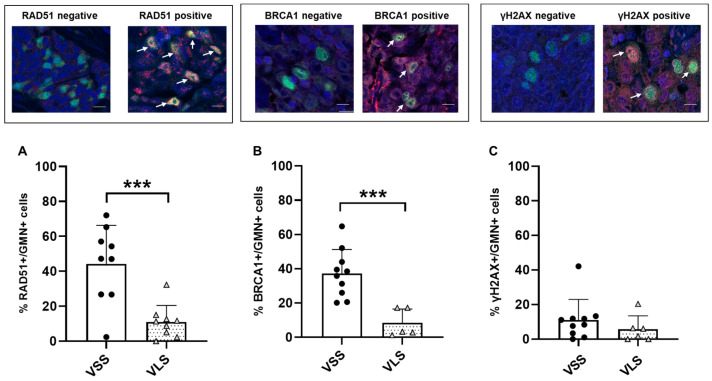
RAD51/BRCA1/γH2AX-foci quantification. Upper panels: Immunofluorescent images of RAD51/BRCA1/γH2AX-foci negative and positive tumor samples. Nuclei (stained with DAPI in blue) of actively proliferating cancer cells (positive for geminin, in green) were evaluated for the absence (negative cells) or presence of RAD51 or BRCA1 or γH2AX- nuclear foci (positive cells, pointed by the white arrows), visible as red dots within the nuclei with a magnification of 60×. Lower panels: Percentage of RAD51+/GMN+ (**A**), BRCA1+/GMN+ (**B**) and γH2AX+/GMN+ (**C**) tumor cells observed in FFPE tumor samples of VSS and VLS MPM patients (***: *p*-value < 0.001; *t*-test). Dots and triagles in all the panels represent each patient’s value.

The downregulation of the DDR score suggested a possible dysregulation/inactivation of DNA repair pathways in patients with a longer survival. *MDC1* was the most downregulated DNA repair gene in VLS compared to VSS patients and considering its multifaceted role in the DDR pathways and specifically in HR [26,27], we applied the recently published RAD51 foci test [22] as a functional assay of HR status. We, thus, evaluated the percentage of RAD51 foci/geminin-positive (RAD51+/GMN+) cells and BRCA1 foci/geminin-positive (BRCA1+/GMN+) cells in the FFPE tumor samples of the same patients (Figure 2). As shown in Figure 2, a statistically significantly lower percentage of RAD51+/GMN+ and BRCA1+/GMN+ tumor cells were observed in VLS than in VSS; a similar percentage of γH2AX+/GMN+ cells were found in both patient subgroups. While no association could be found between RAD51 mRNA expression level and the % of RAD51+/GMN+ cells (Spearman correlation index = 0.1765, *p* = 0.4836), a slight association was found between BRCA1 mRNA expression and the % of BRCA1+/GMN+ cells (Spearman correlation index = 0.5880, *p* = 0.0233) (Supplementary Figure S3). Interestingly enough, all tumors with a low level of RAD51-positive cells, except one case, also displayed a low level of BRCA1-positive cells (Supplementary Table S7). As a whole, these data point to a possible inactivation of HR repair in epithelioid MPM patients displaying longer survival.

## 4. Discussion

MPM is a highly lethal neoplasm that develops in the pleural cavity starting from surface mesothelial cells. In about 80% of the cases, it develops 30–40 years after asbestos exposure, the most recognized environmental-related cause of MPM [28]. Asbestos inhalation causes a chronic inflammation, which induces reactive oxygen species formation and consequently DNA damages and genomic mutations in mesothelial cells that ultimately lead to MPM onset [29,30]. Considering that MPM is generally diagnosed at an advanced stage and is quite refractory to standard chemotherapy, the prognosis of these patients is poor with a with a survival time of 9–18 months [1]. 

Mutations in DNA repair genes and tumor suppressor genes have been reported in MPM (including *BAP1*, *BRCA2*, *CHEK2*, *MLH1*, *MRE11A,* and *PALB2*). Most of the reported genes are involved in specific DNA repair pathways, such as HR, mismatch repair, and nucleotide excision repair [31,32]. The presence of mutations affecting these genes has been associated with an increased OS as compared to MPM patients not bearing such mutations [9]. The improved survival was interpreted, in analogy with what was reported for ovarian cancer patients [33], as a better response to platinum-based therapy, as cisplatin-pemetrexed is the gold standard front-line therapy in MPM [1]. In fact, germ-line mutations in *BAP1*, *BRCA2*, *MLH1*, *MRE11A*, or *PALB2* have been shown in MPM patients with better OS after standard platinum-based chemotherapy than in patients without these variants [9,18,32]. The reported alterations of HR repair genes in MPM patients suggested the possibility to explore the therapeutic potential of PARPi in MPM patients with specific mutations (Clinicaltrials.gov no NTC03531840 and NCT03207347). However, the preliminary data available for the efficacy of PARPi are contrasting. While the combination of cisplatin and PARPi is active in vitro in mesothelioma cells lacking HR repair [34], the published clinical data are contrasting [15,16]. Olaparib efficacy was quite limited in refractory pleural and peritoneal mesothelioma patients, including patients with mutation in DNA repair genes (*BAP1*, *MRE11A*) [15]. Rucaparib demonstrated higher efficacy in a phase II trial, when given to patients with *BAP1*-negative or *BRCA1*-negative mesothelioma with a disease control rate of 58% at 12 weeks (95% CI 37–77; 15 of 26 patients), and at 24 weeks was 23% (9–44; six of 26 patients) [16].

While histology has been reported as a well-known prognostic factor in MPM with epithelioid ones having longer survival than non-epithelioid ones [35], very heterogeneous outcomes have been observed in the former histotype [36]. Our cohort of 54 patients well reflects this heterogeneity with an OS ranging from 1.3 to 106.4 months. No definite molecular and/or biological prognostic biomarkers have been reported, even if some have been suggested (i.e., CTGF-Connective Tissue Growth Factor- protein [37] and the VISTA immune-related protein [38]). Recently, a higher number of B lymphocytes and a prevalence of tertiary lymphoid structures were present in long survivors (>36 months) versus short survivors (>12 months) [36], which has been reported that in a retrospective series of MPM. 

We studied the expression profile of a series of epithelioid MPM patients to find genes associated with OS. Even we found lower *NR4A1*, *NR4A3*, *GRIN2A*, and *FOS* levels in L as compared to S survivors, their correlation with survival was seen only in univariate analysis, strongly limiting its clinical value. 

More interestingly, we observed a decrease in the DDR score from SS to LS, and an even greater difference between VSS and VLS, while all the other 12 pathways’ scores evaluated had an opposite trend. Our group recently published that the RAD51 foci score predicted olaparib sensitivity in a panel of patient-derived ovarian cancer xenografts (the lower the RAD51 foci score, the greater olaparib response) [22]. This test can be considered a read out of a functional test of HR repair and differs from what was reported on the RAD51 foci induction after treatment with DNA damaging agents; it evaluates the number of RAD51 foci in tumor-proliferating cells (geminin-positive cells) control, untreated condition (basal condition, i.e., tumor at diagnosis). This assay has recently been reported to correlate with HR deficiency and predict PARPi response [22]; in addition, it is accurate enough to predict platinum sensitivity in breast cancer [23]. Its validation is, however, under clinical investigation. In our MPM cohort, tumor samples with a low number of RAD51 foci displayed a low number of BRCA1 foci, likely corroborating defects in the HR repair pathway. Interestingly, similar low level of γH2AX foci/geminin-positive cells was observed. This is an intriguing observation as γH2AX is generally associated with increased DNA double-strand breaks, which could be hypothesized in VLS tumor samples with a clear down-regulation of DNA repair pathways. These data contrast with the high percentage of γH2AX foci/geminin-positive cells we observed in ovarian cancers originating from patient-derived xenografts, in which the low number of RAD51 foci predicted olaparib response [22]. However, these data could correlate with the higher genomic instability and higher prevalence of mutations in the TCGA cohort of ovarian carcinomas as compared to mesothelioma [39].

Our findings suggest that VLS MPM patients are enriched in the inactivation of HR repair as the decrease in DNA repair signature and low levels of RAD51 and BRCA1 foci scores using immunofluorescence would suggest. This is the first study analyzing in a functional way the DNA repair in MPM. We were unable to retrieve for all our patients whether and what type of chemotherapy they underwent; thus, we could not explore its role in predicting response to specific treatment in this cohort. 

## 5. Conclusions

Our data strongly support that longer MPM survivors display down-regulation of DDR signature and showed statistically significant lower RAD51 and BRCA1 foci scores as compared to shorter MPM survivors. These data need to be validated in prospective studies where it could be possible to explore both the RAD51 and BRCA1 foci prognostic and predictive roles as determinants of response to chemotherapy. If these data can be validated, we will have an easy, cost-effective test to be routinely used to better tailor chemotherapy in MPM patients.

## Figures and Tables

**Table 1:** MPM patients’ characteristics.

	*n*	%
**Age (years)**		
Median	73	
Range	56–86	
**Gender**		
Female	9	16.7
Male	45	83.3
**Histotype**		
Epitheliod	54	100
**Chemotherapy**		
Yes	23	42.6
No	4	7.4
Unknown	27	50
**Radiotherapy**		
Yes	21	38.9
No	2	3.7
Unknown	31	57.4
**Survival (months)**		
Median (range)	16.9 (1.3–107.7)	
% 1-year survival (95%CI)	68.5%	
% 2-year survival (95%CI)	37.1%	
**Survivors subtype**		
Short	26	48%
Very short	12	22%
Long	28	52%
Very long	12	22%

## Data Availability

Available upon request to the corresponding authors.

## Supplementary Materials

The following supporting information can be downloaded at: https://www.mdpi.com/article/10.3390/cancers15174309/s1, Figure S1: Overall survival (OS) of pleural mesothelioma patients’ cohort analyzed in this study; Figure S2: Gene expression analyses in short and long patient survivors; Figure S3: Correlation between the percentage of RAD51 and BRCA1 foci positive cells and their corresponding mRNA levels; Table S1: List of the primers used for RT- PCR experiments; Table S2: List of the antibodies used for immunofluorescence studies; Table S3: Descriptive statistics of the MPM population under study; Table S4: Treatments of the MPM patients under study; Table S5: Genes associated with MPM patients’ OS and subsequently validated by RT-PCR; Table S6: Log2 fold change of DNA repair genes in VSS versus VLS MPM patients under study; Table S7: MPM tumors with low and high percentage of RAD51 foci positive cell and their corresponding BRCA1 foci levels.

## References

[1] Carbone M., Yang H. (2017). Mesothelioma: Recent highlights. Ann. Transl. Med..

[2] Ahmed M., Flannery A., Mujammil I., Breen D. (2018). Variation in incidence trends of malignant pleural mesothelioma in Europe. Eur. Respir. J..

[3] Davis A., Ke H., Kao S., Pavlakis N. (2022). An Update on Emerging Therapeutic Options for Malignant Pleural Mesothelioma. Lung Cancer.

[4] Rondon L., Fu R., Patel M.R. (2023). Success of Checkpoint Blockade Paves the Way for Novel Immune Therapy in Malignant Pleural Mesothelioma. Cancers.

[5] Knijnenburg T.A., Wang L., Zimmermann M.T., Chambwe N., Gao G.F., Cherniack A.D., Fan H., Shen H., Way G.P., Greene C.S. (2018). Genomic and Molecular Landscape of DNA Damage Repair Deficiency across The Cancer Genome Atlas. Cell Rep..

[6] Malakoti F., Targhazeh N., Abadifard E., Zarezadeh R., Samemaleki S., Asemi Z., Younesi S., Mohammadnejad R., Hadi Hossini S., Karimian A. (2022). DNA repair and damage pathways in mesothelioma development and therapy. Cancer Cell Int..

[7] Panou V., Roe O.D. (2020). Inherited Genetic Mutations and Polymorphisms in Malignant Mesothelioma: A Comprehensive Review. Int. J. Mol. Sci..

[8] Pagliuca F., Zito Marino F., Morgillo F., Della Corte C., Santini M., Vicidomini G., Guggino G., De Dominicis G., Campione S., Accardo M. (2021). Inherited predisposition to malignant mesothelioma: Germline BAP1 mutations and beyond. Eur. Rev. Med. Pharmacol. Sci..

[9] Hassan R., Morrow B., Thomas A., Walsh T., Lee M.K., Gulsuner S., Gadiraju M., Panou V., Gao S., Mian I. (2019). Inherited predisposition to malignant mesothelioma and overall survival following platinum chemotherapy. Proc. Natl. Acad. Sci. USA.

[10] Guo R., DuBoff M., Jayakumaran G., Kris M.G., Ladanyi M., Robson M.E., Mandelker D., Zauderer M.G. (2020). Novel Germline Mutations in DNA Damage Repair in Patients with Malignant Pleural Mesotheliomas. J. Thorac. Oncol..

[11] Sculco M., La Vecchia M., Aspesi A., Pinton G., Clavenna M.G., Casalone E., Allione A., Grosso F., Libener R., Muzio A. (2022). Malignant pleural mesothelioma: Germline variants in DNA repair genes may steer tailored treatment. Eur. J. Cancer.

[12] Konstantinopoulos P.A., Ceccaldi R., Shapiro G.I., D’Andrea A.D. (2015). Homologous Recombination Deficiency: Exploiting the Fundamental Vulnerability of Ovarian Cancer. Cancer Discov..

[13] O’Malley D.M., Krivak T.C., Kabil N., Munley J., Moore K.N. (2023). PARP Inhibitors in Ovarian Cancer: A Review. Target. Oncol..

[14] Panou V., Gadiraju M., Wolin A., Weipert C.M., Skarda E., Husain A.N., Patel J.D., Rose B., Zhang S.R., Weatherly M. (2018). Frequency of Germline Mutations in Cancer Susceptibility Genes in Malignant Mesothelioma. J. Clin. Oncol..

[15] Ghafoor A., Mian I., Wagner C., Mallory Y., Agra M.G., Morrow B., Wei J.S., Khan J., Thomas A., Sengupta M. (2021). Phase 2 Study of Olaparib in Malignant Mesothelioma and Correlation of Efficacy with Germline or Somatic Mutations in BAP1 Gene. JTO Clin. Res. Rep..

[16] A Fennell D., King S., Mohammed A., Branson C., Brookes L., Darlison A.G., Dawson A., Gaba M., Hutka B., Morgan A. (2021). Rucaparib in patients with BAP1-deficient or BRCA1-deficient mesothelioma (MiST1): An open-label, single-arm, phase 2a clinical trial. Lancet Respir. Med..

[17] Betti M., Aspesi A., Ferrante D., Sculco M., Righi L., Mirabelli D., Napoli F., Rondon-Lagos M., Casalone E., Vignolo Lutati F. (2018). Sensitivity to asbestos is increased in patients with mesothelioma and pathogenic germline variants in BAP1 or other DNA repair genes. Genes Chromosomes Cancer.

[18] Baumann F., Flores E., Napolitano A., Kanodia S., Taioli E., Pass H., Yang H., Carbone M. (2015). Mesothelioma patients with germline BAP1 mutations have 7-fold improved long-term survival. Carcinogenesis.

[19] Shrestha R., Nabavi N., Lin Y.Y., Mo F., Anderson S., Volik S., Adomat H.H., Lin D., Xue H., Dong X. (2019). BAP1 haploinsufficiency predicts a distinct immunogenic class of malignant peritoneal mesothelioma. Genome Med..

[20] Thomas M., Dubacq C., Rabut E., Lopez B.S., Guirouilh-Barbat J. (2023). Noncanonical Roles of RAD51. Cells.

[21] Fuh K., Mullen M., Blachut B., Stover E., Konstantinopoulos P., Liu J., Matulonis U., Khabele D., Mosammaparast N., Vindigni A. (2020). Homologous recombination deficiency real-time clinical assays, ready or not?. Gynecol. Oncol..

[22] Guffanti F., Alvisi M.F., Anastasia A., Ricci F., Chiappa M., Llop-Guevara A., Serra V., Fruscio R., Degasperi A., Nik-Zainal S. (2022). Basal expression of RAD51 foci predicts olaparib response in patient-derived ovarian cancer xenografts. Br. J. Cancer.

[23] Pellegrino B., Herencia-Ropero A., Llop-Guevara A., Pedretti F., Moles-Fernandez A., Viaplana C., Villacampa G., Guzman M., Rodriguez O., Grueso J. (2022). Preclinical In Vivo Validation of the RAD51 Test for Identification of Homologous Recombination-Deficient Tumors and Patient Stratification. Cancer Res..

[24] Waggott D., Chu K., Yin S., Wouters B.G., Liu F.F., Boutros P.C. (2012). NanoStringNorm: An extensible R package for the pre-processing of NanoString mRNA and miRNA data. Bioinformatics.

[25] http://media.aiom.it/userfiles/files/doc/LG/2017_LGAIOM_Mesotelioma.pdf.

[26] Ruff S.E., Logan S.K., Garabedian M.J., Huang T.T. (2020). Roles for MDC1 in cancer development and treatment. DNA Repair.

[27] Zimmerlin L., Zambidis E.T. (2020). Pleiotropic roles of tankyrase/PARP proteins in the establishment and maintenance of human naive pluripotency. Exp. Cell Res..

[28] Janes S.M., Alrifai D., Fennell D.A. (2021). Perspectives on the Treatment of Malignant Pleural Mesothelioma. N. Engl. J. Med..

[29] Donaldson K., Murphy F.A., Duffin R., Poland C.A. (2010). Asbestos, carbon nanotubes and the pleural mesothelium: A review of the hypothesis regarding the role of long fibre retention in the parietal pleura, inflammation and mesothelioma. Part. Fibre Toxicol..

[30] Xu A., Wu L.J., Santella R.M., Hei T.K. (1999). Role of oxyradicals in mutagenicity and DNA damage induced by crocidolite asbestos in mammalian cells. Cancer Res..

[31] Hiltbrunner S., Mannarino L., Kirschner M.B., Opitz I., Rigutto A., Laure A., Lia M., Nozza P., Maconi A., Marchini S. (2021). Tumor Immune Microenvironment and Genetic Alterations in Mesothelioma. Front. Oncol..

[32] Betti M., Casalone E., Ferrante D., Aspesi A., Morleo G., Biasi A., Sculco M., Mancuso G., Guarrera S., Righi L. (2017). Germline mutations in DNA repair genes predispose asbestos-exposed patients to malignant pleural mesothelioma. Cancer Lett..

[33] Chiappa M., Guffanti F., Bertoni F., Colombo I., Damia G. (2021). Overcoming PARPi resistance: Preclinical and clinical evidence in ovarian cancer. Drug Resist. Updat..

[34] Borchert S., Wessolly M., Schmeller J., Mairinger E., Kollmeier J., Hager T., Mairinger T., Herold T., Christoph D.C., Walter R.F.H. (2019). Gene expression profiling of homologous recombination repair pathway indicates susceptibility for olaparib treatment in malignant pleural mesothelioma in vitro. BMC Cancer.

[35] Wang S., Ma K., Chen Z., Yang X., Sun F., Jin Y., Shi Y., Jiang W., Wang Q., Zhan C. (2018). A Nomogram to Predict Prognosis in Malignant Pleural Mesothelioma. World J. Surg..

[36] Mannarino L., Paracchini L., Pezzuto F., Olteanu G.E., Moracci L., Vedovelli L., De Simone I., Bosetti C., Lupi M., Amodeo R. (2022). Epithelioid Pleural Mesothelioma Is Characterized by Tertiary Lymphoid Structures in Long Survivors: Results from the MATCH Study. Int. J. Mol. Sci..

[37] Ohara Y., Enomoto A., Tsuyuki Y., Sato K., Iida T., Kobayashi H., Mizutani Y., Miyai Y., Hara A., Mii S. (2020). Connective tissue growth factor produced by cancer-associated fibroblasts correlates with poor prognosis in epithelioid malignant pleural mesothelioma. Oncol. Rep..

[38] Alcala N., Mangiante L., Le-Stang N., Gustafson C.E., Boyault S., Damiola F., Alcala K., Brevet M., Thivolet-Bejui F., Blanc-Fournier C. (2019). Redefining malignant pleural mesothelioma types as a continuum uncovers immune-vascular interactions. eBioMedicine.

[39] Fuso Nerini I., Roca E., Mannarino L., Grosso F., Frapolli R., D’Incalci M. (2020). Is DNA repair a potential target for effective therapies against malignant mesothelioma?. Cancer Treat. Rev..

