# Supplementary material for: Epithelioid Mesothelioma Patients with Very Long Survival Display Defects in DNA Repair
Source: Cancers (Basel). 2023 Aug 29;15(17):4309. doi: 10.3390/cancers15174309 (PMC10486625; doi:10.3390/cancers15174309)
Supplement: Supplementary file 1 [file cancers-15-04309-s001.zip › cancers-2488945-supplementary.pdf]

Supplementary data.

Legend to supplementary figures.

Supplementary Figure S1. Overall survival (OS) of pleural mesothelioma patients' cohort analyzed in this study.

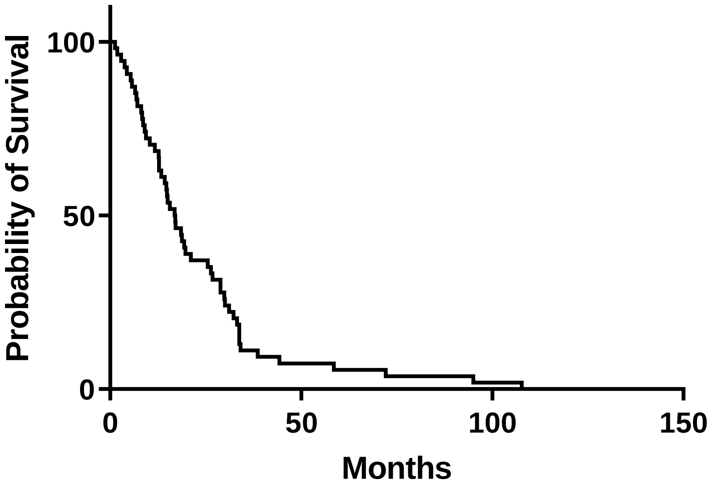

Supplementary Figure S2. Gene expression analyses in short and long patient survivors. Panel A) Nanostring Volcano plot displaying each genes' −log10(p-value) and log2 fold change. Statistically significant differentially expressed genes in long vs short survivors fall above the horizontal lines, that indicate the various *p-value* thresholds. The most statistically significant mRNAs are labelled in blue in the plot. Panel B) RT-PCR validation of the eight most differentially expressed genes in long and short survivors.

A

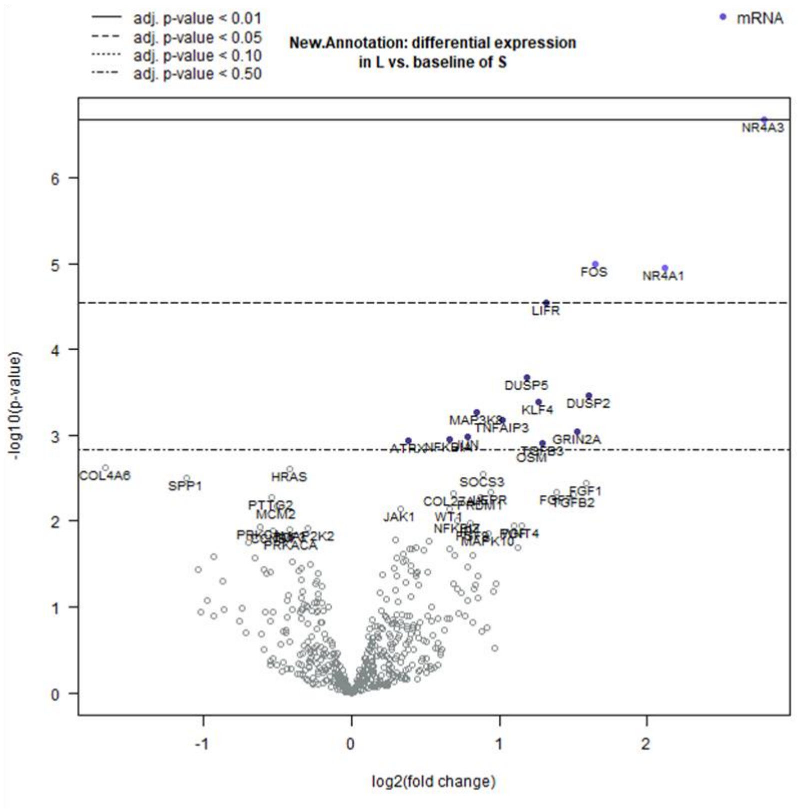

B

Group	gene	mean CT	SD	FC	p value	gene	mean CT	SD	FC	p value
	FOS					LS6M				
SS		4.7	1.8	4	***		7.4	3.7	0.8	ns
LS		2.7	1.8				7.7	2.6		
	GRIN2A					TCF3				
SS		12.5	2.3	4.6	***		5.9	0.96	0.6	ns
LS		10.3	1.5				6.7	2.7		
	NR4A1					KMD5C				
SS		6.7	1.9	2.6	*		5.8	0.97	0.5	ns
LS		5.3	2.4				6.7	3.9		
	NR4A3					GZMB				
SS		9.3	3.2	5.3	***		9.2	1.6	1.7	ns
LS		6.9	2.8				8.4	1.7		

SS: short survivors; LS: long survivors, CT: cycle threshold; SD; standard deviation

Supplementary Figure S3. Correlation between the percentage of RAD51 and BRCA1 foci positive cells and their corresponding mRNA levels. Panel A) Correlation between RAD51+/GMN+ cells and their RAD51 mRNA levels. Panel B) Correlation between the percentage of BRCA1+/GMN+ cells and their BRCA1 mRNA levels. Both analyses were done only in VSS and VLS as only in these patients the quantification of RAD51 and BRCA1 foci was done.

A

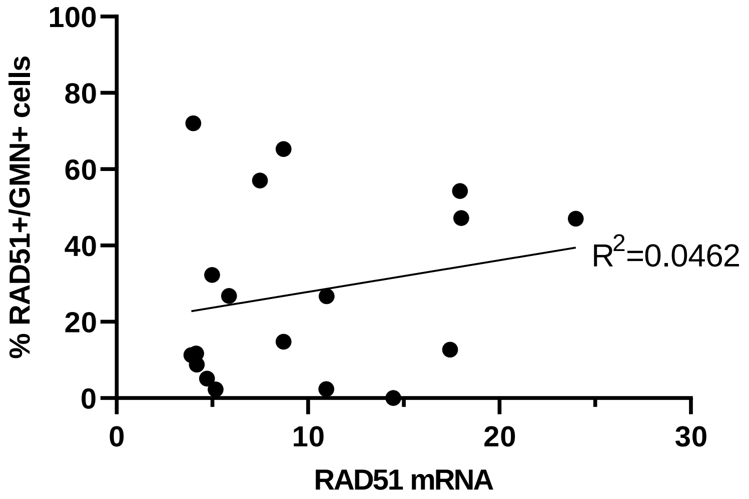

B

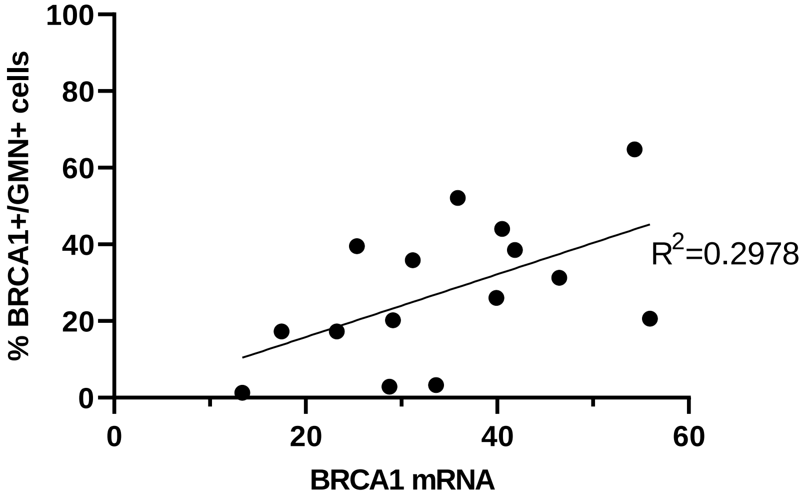

Supplementary Table S1. List of the primers designed for RT-PCRs.

Gene	Primer Forward	Primer Reverse
NR4A1	ACATGTGAGGGCTGCAAGG	CAGTCCTTGTTAGCCAGGCA
NR4A3	CTACGGCGTGCGAACCTG	CGTCTCTTGTCTACTGGGCA
GRIN2A F	CTACGGGCAGATGGAGAGG	ACCTGGTAGCCTTCCTCAGT
FOS	TACTACCACTCACCCGCAGA	CGTGGGAATGAAGTTGGCAC
TCF3	CGCCTATGCCTCCTTCGG	CTGTTGAGGGCCAGCTCG
KMD5C	TGGCCGACTCCTTTAAAGCT	CCAGCCTCCAGAACTCCTTC
LS6M	AAGCAAATCATCGGACGACC	TGCTCCAGGGCTATATTCATGT
GZMB	CTTCTGCTGGCCTTCCTCC	GCTTGGCCTCATGTCCCC

Supplementary Table S2. List of antibodies used for immunofluorescence studies.

Primary antibodies	Supplier	Species	Type	Dilution	Cat #
RAD51	Abcam	Rabbit	Monoclonal	1:1000	ab133534
BRCA1	Santa Cruz Biotechnologies	Mouse	Monoclonal	1:50	sc-6954
γH2AX	Millipore- Merk	Mouse	Monoclonal	1:200	05-636
Geminin	ProteinTech Group	Rabbit	Policlonal	1:400	10802-1-AP
Geminin	Novocastra	Mouse	Monoclonal	1:100	Geminin-L-CE
**Secondary antibodies (Thermo Fisher Scientific)**		**Species**	**Type**	**Dilution**	**Cat #**
Alexa fluor 488		goat-anti mouse	Policlonal	1:500	A-11001
Alexa fluor 488		goat-anti rabbit	Policlonal	1:500	A-32731
Alexa fluor 568		goat-anti mouse	Policlonal	1:500	A-11004
Alexa fluor 568		goat-anti rabbit	Policlonal	1:500	A-11011

Supplementary Table S3. Descriptive statistics of the MPM population under study.

	Total population	Short survivors	Long survivors
Number of values	54	26	28
Minimum (months)	1.3	1.3	16.9
25% Percentile	8.9	5.625	20.05
Median (months)	16.95	8.8	29.95
75% Percentile	30.28	12.95	34.03
Maximum (months)	107.7	15.6	107.7
Range	106.4	14.3	90.8
10% Percentile	4.9	2.6	17.09
90% Percentile	41.45	14.93	74.39
95% CI of median			
Actual confidence level	95.98%	97.10%	96.43%
Lower confidence limit	12.8	6.5	25.5
Upper confidence limit	25.5	12.8	33.8
Mean (months)	22.79	9.142	35.47
Std. Deviation	20.97	4.383	22.34
Std. Error of Mean	2.853	0.8596	4.221
Lower 95% CI of mean	17.07	7.372	26.81
Upper 95% CI of mean	28.52	10.91	44.13

Supplementary Table S4. Treatments of the MPM patients under study.

(no pts)	**Surgery (%)**	**RT (%)**	**CT (%)**	**RT+CT (%)**
**SS (26)**	**18 (69%)**	**12 (46%)**	**11 (42%)**	**11 (42%)**
**LS (28)**	**20 (71%)**	**9 (32%)**	**9 (32%)**	**6 (21%)**
**VSS (12)**	**8 (67%)**	**5 (42%)**	**4 (33%)**	**4 (33%)**
**VLS (10)**	**7 (70%)**	**4 (40%)**	**4 (70%)**	**3 (30%)**

RT: radiotherapy; CT: chemotherapy, generally consisting of a platinum (cisplatin and carboplatin)-based therapy in association with pemetrex, based on the Italian AIOM (Italian Association of Medical Oncology) (http://media.aiom.it/userfiles/files/doc/LG/2017_LGAIOM_Mesotelioma.pdf) and international (DOI: 10.1093/annonc/mdv199)guidelines.

Supplementary Table S5. Genes associated with MPM patients' OS and subsequently validated by RT-PCR.

gene	exp(coef)	exp(-coef)	lower .95	upper .95	z	Pr(>|z|)	fdr
NR4A3	0.74	1.35	0.63	0.88	-3.5025	0.0005	0.0374
FOS	0.70	1.43	0.58	0.85	-3.5856	0.0003	0.0374
GRIN2A	0.65	1.53	0.51	0.84	-3.4038	0.0007	0.0402
NR4A1	0.74	1.35	0.62	0.89	-3.2567	0.0011	0.0457

Supplementary Table S6. Log2 fold change of DNA repair genes in VSS versus VLS MPM patients under study.

Probe Label	Log2 fold change	std error (log2)	Lower confidence limit (log2)	Lower confidence limit (log2)
XPA-mRNA	1	33	355	165
POLR2D-mRNA	-401	14	-675	-127
ERCC6-mRNA	-585	245	-107	-104
NBN-mRNA	-38	188	-748	-111
PCNA-mRNA	-47	238	-938	-303
FANCC-mRNA	344	18	-89	696
PRKDC-mRNA	-535	293	-111	395
POLD4-mRNA	-408	231	-862	455
UBE2T-mRNA	-554	316	-117	655
XRCC4-mRNA	-674	399	-146	107
FANCB-mRNA	-158	109	-372	554
FEN1-mRNA	-751	529	-179	285
RAD50-mRNA	509	45	-373	139
H2AFX-mRNA	-295	285	-854	264
RPS27A-mRNA	-139	149	-431	152
RFC3-mRNA	309	345	-368	986
FANCF-mRNA	36	41	-443	116
MAD2L2-mRNA	-202	23	-653	25
GTF2H3-mRNA	171	209	-239	581
POLB-mRNA	-163	22	-595	269
ATM-mRNA	175	289	-391	741
MDC1-mRNA	-833	142	-361	194
MGMT-mRNA	-167	29	-735	401
BRCA1-mRNA	-63	13	-318	192
FANCA-mRNA	-41	94	-225	143
BRIP1-mRNA	-458	109	-259	167
FANCL-mRNA	403	975	-151	231
NTHL1-mRNA	-496	123	-291	192
POLR2H-mRNA	-63	172	-4	274
ALKBH2-mRNA	-376	116	-265	19
MNAT1-mRNA	288	94	-155	213
POLR2J-mRNA	-375	137	-306	231
LIG4-mRNA	321	119	-201	266
ERCC2-mRNA	-288	108	-241	183
BRCA2-mRNA	375	144	-245	32
POLD1-mRNA	-371	194	-417	343
MUTYH-mRNA	199	105	-187	227
RFC4-mRNA	524	285	-505	61
ALKBH3-mRNA	145	102	-185	214
DDB2-mRNA	211	209	-388	43
RAD52-mRNA	191	248	-467	505
UBB-mRNA	-153	212	-432	401
FANCE-mRNA	471	121	-233	242
C19orf40-mRNA	554	143	-274	285
FANCG-mRNA	545	174	-335	346

Supplementary Table S7. MPM tumors with low and high percentage of RAD51 foci positive cell and their corresponding BRCA1 foci levels.

	Low RAD51+/GMN+ cells		High RAD51+/GMN+ cells	
	**% RAD51+/GMN+ cells**	**% BRCA1+/GMN+ cells**	**% RAD51+/GMN+ cells**	**% BRCA1+/GMN+ cells**
	2.3	3.3	26.7	20.6
	2.4	64.8	26.8	26.0
	8.8	1.3	32.3	17.3
	11.7	17.3	47.0	31.3
	12.7	2.9	47.2	44.0
			54.3	52.1
			57.0	35.9
			65.3	39.5
			72.0	20.2
Median	*8.80*	*3.30*	*47.20*	*31.30*
Min-max	*2.3 - 12.7*	*1.3 - 64.8*	*26.7 - 47.2*	*17.3 - 52.1*
